# *In vitro* activity of sulbactam in combination with other antimicrobial agents against extensively drug-resistant *Acinetobacter baumannii*

**DOI:** 10.1128/spectrum.01379-25

**Published:** 2025-08-12

**Authors:** Anupop Jitmuang, Surapee Tiengrim, Visanu Thamlikitkul, Pornpan Koomanachai

**Affiliations:** 1 Division of Infectious Diseases and Tropical Medicine, Department of Medicine, Faculty of Medicine Siriraj Hospital, Mahidol University 65106, Bangkok, Thailand; University of Manitoba, Winnipeg, Canada

**Keywords:** *Acinetobacter baumannii*, extensively drug resistant, synergy testing, sulbactam

## Abstract

Extensively drug-resistant (XDR) *Acinetobacter baumannii* infection has significant challenges due to limited treatment options. Although sulbactam (SUL) shows *in vitro* effectiveness against XDR *A. baumannii*, the efficacy of SUL-based combinations remains unclear. This investigation aimed to delineate the *in vitro* activity of SUL combined with various antimicrobial agents against XDR *A. baumannii*. Sixty-two clinical isolates of XDR *A. baumannii* were tested for minimal inhibitory concentrations (MICs) of SUL, amikacin (AMI), ciprofloxacin, colistin (COL), fosfomycin (FOS), gentamicin, meropenem (MER), rifampicin (RIF), sitafloxacin (SIT), and tigecycline (TIG) using broth microdilution. The checkerboard method, employing the fractional inhibitory concentration index, assessed *in vitro* synergy between the SUL-based combination. Time–kill analyses of selected isolates were conducted to measure log_10 _colony-forming unit per milliliter growth changes over 24 hours between individual and combined agents. The SUL MICs ranged from <4 to 256 mg/L, with an MIC_50_ of 64 mg/L. MIC ranges were lower for TIG (0.12–4.0 mg/L) and COL (0.5–2.0 mg/L), but higher for FOS (64–>512 mg/L). Synergism was evident in the combinations of SUL/FOS (41.9%), SUL/AMI (19.3%), SUL/MER (17.7%), SUL/RIF (14.5%), SUL/TIG (12.9%), SUL/COL (6.5%), and SUL/SIT (4.8%). Only 1.6%–3.2% of the combinations exhibited antagonism. In the time–kill assays, a combination of SUL/FOS/AMI/MER exhibited sustained bactericidal activity at 24 hours against the two isolates, whereas two- and three-agent combinations showed varying degrees of synergism. Combining SUL with available antimicrobial agents had varying degrees of synergistic effect against XDR *A. baumannii*. Notably, the clinical utility of SUL-based combination therapy for XDR *A. baumannii* infections requires further exploration.

**IMPORTANCE:**

This study evaluated the *in vitro* effectiveness of SUL combined with other antibiotics against XDR *Acinetobacter baumannii*. Sixty-two clinical isolates were tested using broth microdilution and checkerboard methods. The SUL MIC_50_ was 64 mg/L, with tigecycline and colistin showing lower MIC ranges and higher fosfomycin. Synergistic activity was most notable with SUL/fosfomycin (41.9%), followed by SUL/amikacin (19.3%) and SUL/meropenem (17.7%). Antagonism was rare (1.6%–3.2%). Time–kill assays showed that the four-drug combination of SUL/fosfomycin/amikacin/meropenem had sustained bactericidal activity over 24 hours. While SUL-based combinations showed variable synergy, further studies are needed to determine their clinical potential.

## INTRODUCTION

*Acinetobacter baumannii*, a critical gram-negative pathogen, is a predominant cause of hospital-associated infections in Thailand (1–4). Notoriously known for its capacity to develop resistance against multiple classes of antimicrobial agents, the extensively drug-resistant (XDR) *A. baumannii* poses significant therapeutic dilemmas. XDR *A. baumannii* is resistant to nearly all antimicrobial agents, and susceptibility is limited to one or two options (5), leading to challenging treatment scenarios and elevated mortality rates. An investigation of *A. baumannii* across 13 countries in the Asia–Pacific region shows that 71.7% of *A. baumannii* strains are resistant to multiple antimicrobials, including carbapenems (6). The rate of carbapenem-resistant *A. baumannii* varies, with lower levels in Japan (2.8%) and Australia (6.5%) and much higher rates exceeding 80% in several countries, including China (80.6%), Thailand (83.0%), Pakistan (85.0%), India (87.2%), and Korea (88.0%) (6). Additionally, the rate of carbapenem-resistant *A. baumannii* has been significantly increasing in Latin America and Europe, while it has grown moderately in the United States (7). From these epidemiological studies, XDR *A. baumannii* has emerged as a highly significant pathogen in healthcare settings worldwide. In Thailand, colistin is the frontline antimicrobial for XDR *A. baumannii* infections. Nonetheless, treatments involving colistin, either as monotherapy or in combination with fosfomycin (FOS), meropenem (MER), rifampin, or sitafloxacin (SIT), are associated with mortality rates ranging from 40% to 60% (8–13). Tigecycline (TIG), a derivative of tetracycline, has been subjected to limited clinical trials for these infections (14), while cefiderocol, a new agent showing good *in vitro* activity against XDR *A. baumannii* (15, 16), is not yet available in Thailand.

Sulbactam (SUL), a synthetic beta-lactam derivative, has demonstrated notable *in vitro* activity against *A. baumannii* (17, 18). Historically, ampicillin-sulbactam or cefoperazone-sulbactam has been combined to combat beta-lactamase-producing bacteria, while a new sulbactam-based agent—durlobactam-sulbactam—has recently become a preferred treatment for XDR *A. baumannii* infections, as recommended by the Infectious Diseases Society of America (IDSA) (14, 19). Durlobactam, a novel beta-lactamase inhibitor, can inhibit several classes of beta-lactamases, including class D enzymes, which allow sulbactam to effectively reach penicillin-binding protein targets (20, 21). However, as a resource-limited country, Thailand currently does not have access to this novel agent. Fortunately, sulbactam is now available in Thailand as an intravenous single-agent formulation, emerging as a potential adjunctive treatment option for XDR *A. baumannii* infections.

IDSA 2024 recommends a total sulbactam dose of 9 g daily, such as 18 g of ampicillin–9 g of sulbactam co-formulation, in combination with other antimicrobial agents such as colistin, minocycline, or tigecycline for XDR *A. baumannii* infections (14). However, a total sulbactam dose of 9–12 g/day is only sufficient for treating XDR *A. baumannii* isolates with minimal inhibitory concentrations (MICs) of 16–32 mg/L, as reported by Jaruratanasirikul et al*.* (22). Interestingly, in Thailand, recent XDR *A. baumannii* isolates causing severe infections have shown a significant increase in MICs, ranging from 32 to >256 mg/L (22–24). Therefore, the total sulbactam dose recommended by the IDSA may not achieve *in vitro* effectiveness against isolates with high MICs of >32 mg/L, based on pharmacokinetic studies (22, 25). The safety and efficacy of doses exceeding 12 g/day of sulbactam have not been fully studied, and it remains unclear whether the benefits of higher dosing outweigh the risks of toxicity from sulbactam and co-administered agents like ampicillin.

Sulbactam is now used as an adjunct antimicrobial option to treat XDR *A. baumannii *infection in Thailand. Preliminary *in vitro* studies from our team indicate that sulbactam, in combination with colistin, does not exhibit synergistic effects against XDR *A. baumannii* isolates (24). However, the potential of various sulbactam-based combinations has yet to be fully elucidated, and the current evidence is limited (14, 26). Consequently, this study aimed to explore and identify effective combinations of SUL with nine available antimicrobial agents, such as amikacin (AMI), ciprofloxacin (CIP), colistin (COL), FOS, gentamicin (GEN), MER, rifampicin (RIF), SIT, and TIG to optimize *in vitro* activity against XDR *A. baumannii* isolates.

## RESULTS

### MIC determination for SUL and other agents against XDR *A. baumannii* isolates

The MICs of SUL and nine other antimicrobial agents were determined against 62 XDR *A. baumannii* isolates, as detailed in Table 1 and Table S1. The MIC range for SUL varied from ≤4 to 256 mg/L, with MIC_50_ and MIC_90_ values of 64 and 128 mg/L, respectively. The other agents exhibited MICs ranging from low to very high. COL, SIT, and TIG had consistently low MICs, with COL ranging from 0.5 to 2.0 mg/L (MIC_50_: 1 mg/L, MIC_90_: 1 mg/L); SIT ranging from ≤0.12 to 4.0 mg/L (MIC_50_: 1 mg/L, MIC_90_: 4 mg/L); and TIG ranging from 0.12 to 4.0 mg/L (MIC_50_: 1 mg/L, MIC_90_: 1 mg/L). In contrast, agents such as AMI, CIP, GEN, and RIF displayed variable MIC ranges with increased MIC_50_ and MIC_90_ values. Notably, FOS and MER demonstrated significantly high MIC levels. All the isolates exhibited intermediate susceptibility to COL, while limited susceptibility was noted for AMI (12.3%), CIP (1.5%), and GEN (10.8%). No isolates were susceptible to MER. Of these isolates, 50.8% exhibited MICs of ≤1 mg/L, while 89.2% had MICs of ≤2 mg/L to SIT.

**TABLE 1:** Minimal inhibitory concentrations of sulbactam and nine additional antimicrobial agents against 62 extensively drug-resistant *A. baumannii* isolates^*a*^

Antimicrobial agent	MIC (mg/L)			MIC breakpoints according to the CLSI criteria	No. of isolates (%)
	Range	MIC_50_	MIC_90_		
Sulbactam	≤4–256	64	128	na	na
Amikacin	0.25–128	64	64	≤16 (S)	8 (12.3)
Ciprofloxacin	0.5–>32	>32	>32	≤1 (S)	1 (1.5)
Colistin	0.5–2.0	1	1	≤2 (I)^*b*^	62 (100)^*b*^
Fosfomycin	64–>512	512	>512	na	na
Gentamicin	≤0.12–>32	>32	>32	≤4 (S)	7 (10.8)
Meropenem	16–>256	128	256	≤2 (S)	0
Rifampicin	1–128	8	128	na	na
Sitafloxacin	≤0.12–4.0	1	4	≤1 (S)	33 (50.8)
				≤2 (S)^*c*^	58 (89.2)
Tigecycline	0.12–4.0	1	1	na	na

^*a*^ CLSI, Clinical and Laboratory Standards Institute; I, intermediate; MIC, minimal inhibitory concentration; na, not available; S, susceptible.

^*b*^ Interpretative breakpoint for susceptibility to colistin has been omitted; only intermediate (MICs ≤2 mg/L) and resistant (MICs ≥4 mg/L) breakpoints as per the CLSI guideline M100, 2020, are employed (27).

^*c*^ MICs for sitafloxacin ≤2 mg/L are defined as susceptible according to Rodjun et al. (28).

### Synergy testing outcomes using the checkerboard method

Synergistic effects determined by the checkerboard method revealed that SUL combined with FOS had a synergistic effect (fractional inhibitory concentration index [FICI] ≤0.5) on 26 (41.9%) of the isolates. Other combinations had varying degrees of synergy: SUL/AMI in 12 (19.3%), SUL/MER in 11 (17.7%), SUL/RIF in 9 (14.5%), SUL/TIG in 8 (12.9%), SUL/COL in 4 (6.5%), SUL/SIT in 3 (4.8%), and SUL/GEN in 2 (3.2%) isolates (Table 2). The combination of SUL/MER had an additive effect on 39 (62.9%) isolates. Additive effects in other combinations affected 8 (12.9%) to 30 (48.4%) of the isolates. Notably, several combinations, especially with AMI, CIP, COL, GEN, SIT, and TIG, exhibited indifference. Antagonistic interactions were noted in a few cases: for the SUL/CIP combination in 2 (3.2%) isolates, for the SUL/RIF combination, in 1 (1.6%) isolate and for the SUL/SIT combination, in 1 (1.6%) isolate.

**TABLE 2:** Proportions of fractional inhibitory concentration indices of individual sulbactam-based combinations against 62 extensively drug-resistant *A. baumannii* isolates determined by the checkerboard microdilution method^*a*^

Sulbactam combined with the studied agent	No. of isolates (%)			
	FICI category			
	Synergistic	Additive	Indifferent	Antagonistic
Sulbactam/amikacin	12 (19.3)	13 (21.0)	37 (59.7)	0
Sulbactam/ciprofloxacin	0	8 (12.9)	52 (83.9)	2 (3.2)
Sulbactam/colistin	4 (6.5)	24 (38.7)	34 (54.8)	0
Sulbactam/fosfomycin	26 (41.9)	25 (40.3)	11 (17.7)	0
Sulbactam/gentamicin	2 (3.2)	29 (46.8)	31 (50.0)	0
Sulbactam/meropenem	11 (17.7)	39 (62.9)	12 (19.3)	0
Sulbactam/rifampicin	9 (14.5)	30 (48.4)	22 (35.5)	1 (1.6)
Sulbactam/sitafloxacin	3 (4.8)	28 (45.2)	30 (48.4)	1 (1.6)
Sulbactam/tigecycline	8 (12.9)	20 (32.3)	34 (54.8)	0

^*a*^ FICI, fractional inhibitory concentration index.

### Time–kill method evaluation of antimicrobial agents against selected XDR *A. baumannii*

**Fig 1:**
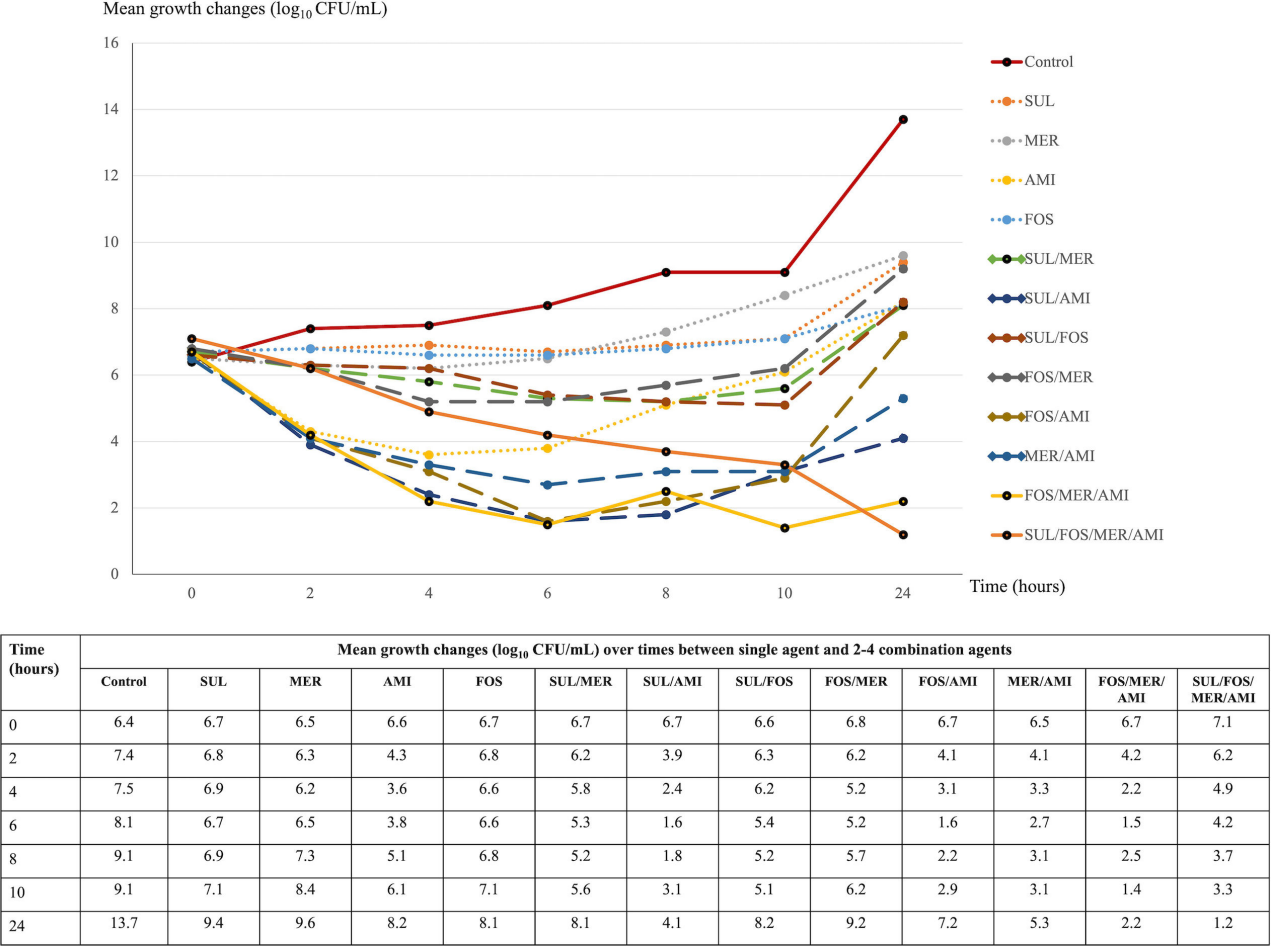
Time–kill assay: mean growth changes (log_10_ CFU/mL) over time from the initial inoculum (0–24 hours) for single and two to four agent combinations against the XDR *A. baumannii* isolate KO107. Abbreviations: AMI, amikacin; CFU, colony-forming unit; FOS, fosfomycin; MER, meropenem; SUL, sulbactam.

**Fig 2:**
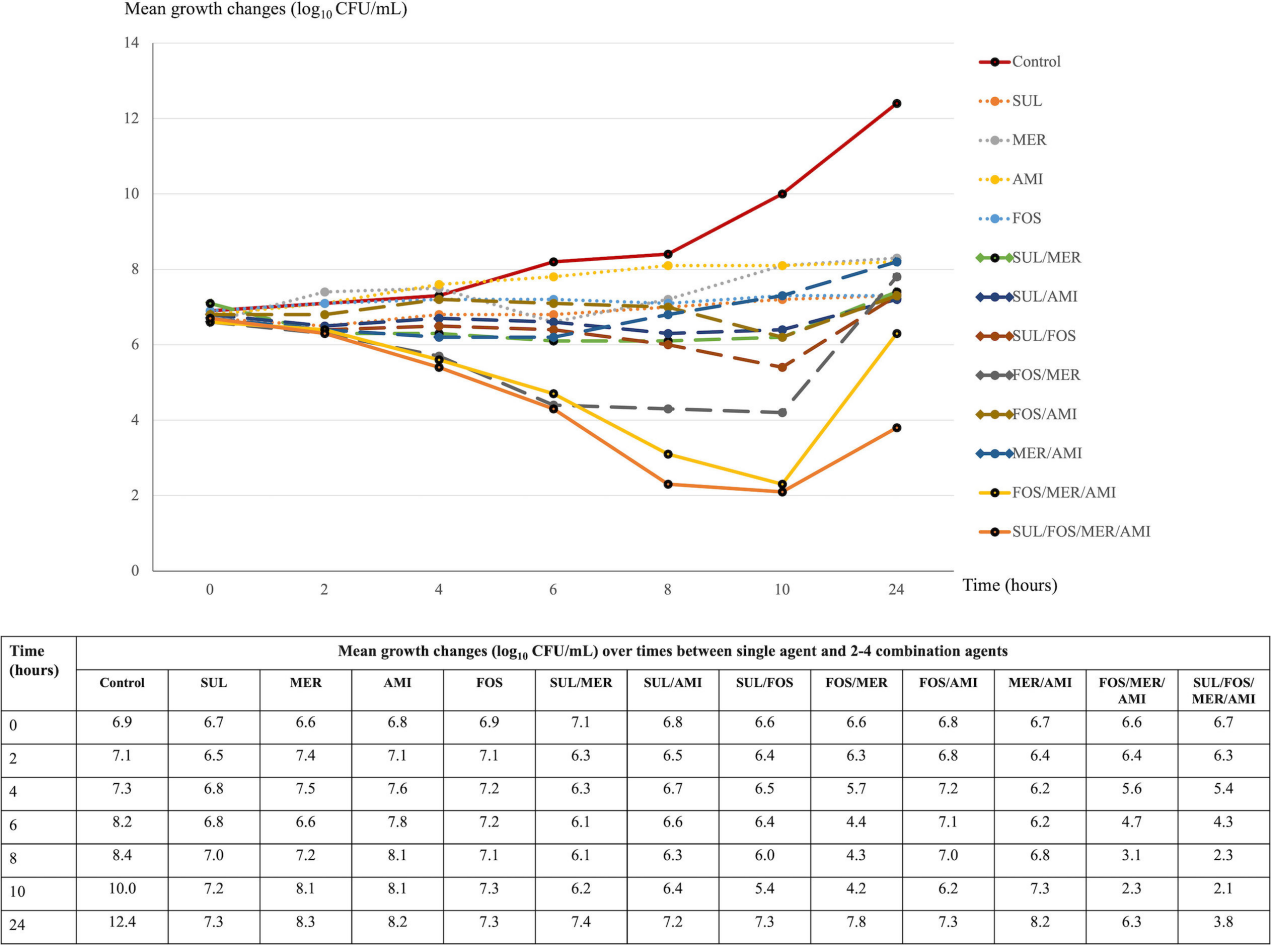
Time–kill assay: mean growth changes (log_10_ CFU/mL) over time from the initial inoculum (0–24 hours) for single and two to four agent combinations against the XDR *A. baumannii* isolate SiUA6. Abbreviations: AMI, amikacin; CFU, colony-forming unit; FOS, fosfomycin; MER, meropenem; SUL, sulbactam.

**Fig 3:**
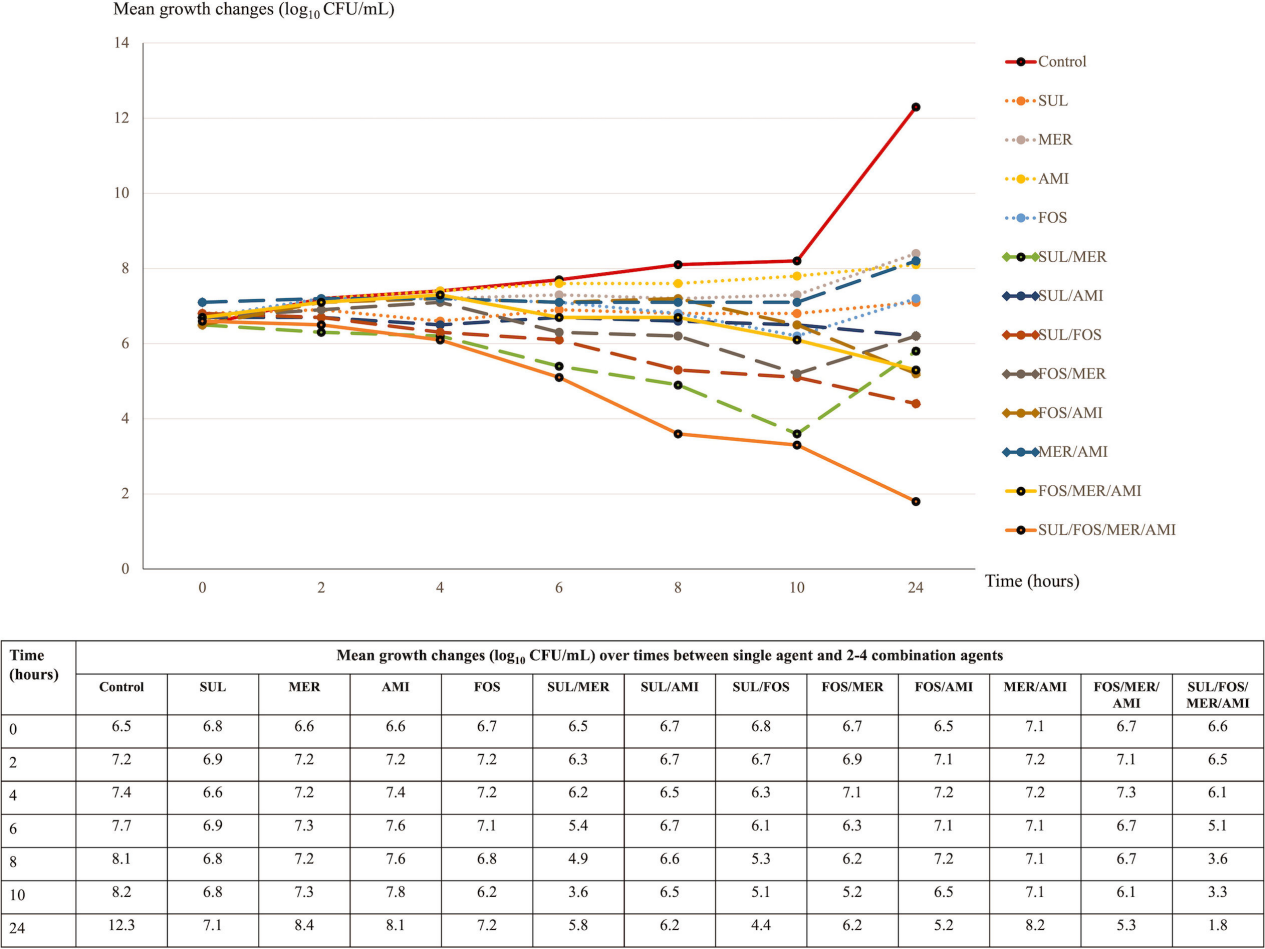
Time–kill assay: mean growth changes (log_10_ CFU/mL) over time from the initial inoculum (0–24 hours) for single and two to four agent combinations against the XDR *A. baumannii* isolate SiPA14. Abbreviations: AMI, amikacin; CFU, colony-forming unit; FOS, fosfomycin; MER, meropenem; SUL, sulbactam.

The study selected AMI, FOS, MER, and SUL for their notable *in vitro* synergistic potential, as identified in checkerboard assays. These agents were used to assess bactericidal activity over 24 hours against three distinct XDR *A. baumannii* isolates using the time–kill method. The selected isolates were KO107 (MICs of SUL 64 mg/L, AMI 64 mg/L, FOS 512 mg/L, and MER 256 mg/L), SiUA6 (MICs of SUL 64 mg/L, AMI 1 mg/L, FOS 512 mg/L, and MER 256 mg/L), and SiPA14 (MICs of SUL 128 mg/L, AMI 64 mg/L, FOS 512 mg/L, and MER 128 mg/L). Synergistic effects determined by the checkerboard method were observed for SUL combinations against the KO107 and SiUA6 isolates but not against the SiPA14 isolate. Growth alterations are illustrated in Fig. 1 to 3. At concentrations of 0.25× MICs, the AMI, FOS, MER, and SUL monotherapies had no bactericidal effects on these isolates within 24 hours.

### Bactericidal activity at different time points

For isolate KO107, combinations of SUL/AMI, FOS/AMI, and MER/AMI significantly reduced bacterial levels at 6 and 8 hours, with regrowth observed by 24 hours (Fig. 1). In the case of SiUA6 and SiPA14, these combinations demonstrated minimal bactericidal activity at 6 and 8 hours (Fig. 2 and 3). A triple combination of FOS/MER/AMI effectively lowered the bacterial count of KO107 cells, maintaining this reduction for up to 24 hours (Fig. 1). This regimen exhibited moderate activity against SiUA6 at 8–10 hours (Fig. 2) and had a limited impact on SiPA14 (Fig. 3). The four-agent combination of SUL/FOS/AMI/MER resulted in a substantial reduction of bacterial growth (3–4 log_10_ colony-forming unit [CFU]/mL) at 8–10 hours for all three isolates. This regimen continued to suppress bacterial growth in the KO107 (Fig. 1) and SiPA14 (Fig. 3) isolates at 24 hours, but moderate regrowth was observed in the SiUA6 strain (Fig. 2).

### Synergistic effects at 24 hours

Compared with those of individual agents, the combinations of SUL/AMI, MER/AMI, and FOS/MER/AMI achieved a growth reduction of ≥2 log_10_ CFU/mL (indicative of synergy) at 24 hours in isolate KO107 (Fig. 1). The SUL/FOS and FOS/AMI combinations also significantly reduced the growth of the SiPA14 isolate after 24 hours (Fig. 3). However, dual- and triple-agent combinations did not significantly reduce the growth of SiUA6 cells at the 24 hour time point (Fig. 2).

## DISCUSSION

In this investigation of 62 XDR *A. baumannii* isolates, SUL exhibited a broad range of MICs, spanning ≤4–256 mg/L. The median (MIC_50_) and high percentile (MIC_90_) values were recorded at 64 and 128 mg/L, respectively, which are notably higher than the SUL MICs reported in other studies (29, 30). Synergistic effects were observed when SUL was combined with FOS, AMI, or MER, affecting 41.9%, 19.3%, and 17.7%, respectively, of the isolates. Time–kill assays particularly highlighted the synergistic effects of SUL- and non-SUL combinations against the KO107 and SiPA14 strains. However, at 0.25× MICs, most dual- and triple-agent combinations did not exhibit enhanced bactericidal activity at 24 hours, with the notable exception of the four-agent combination of SUL/FOS/AMI/MER.

According to the study, COL, TIG, and SIT have lower MICs against the XDR *A. baumannii* isolates than other tested agents. According to the Clinical and Laboratory Standards Institute (CLSI) interpretative breakpoints, all study isolates exhibited intermediate susceptibility to COL (27). The MIC of COL has varied considerably across studies (0.25–16.0 mg/L) (31–34), while TIG MICs have been consistently unchanged over recent years (0.25–4.0 mg/L) (33, 35, 36). Interestingly, using the MIC breakpoints suggested by Rodjun et al., we found that 50.8% of the isolates were susceptible to SIT at a concentration of ≤1 mg/L, while 89.2% were susceptible to this agent at a concentration of ≤2 mg/L (28). SIT, a newer fluoroquinolone, has demonstrated good *in vitro* activity against highly drug-resistant *A. baumannii* isolates, with more than 90% of the strains remaining susceptible to this agent (28, 36, 37). In contrast, FOS exhibits a high MIC against the isolates, suggesting limited efficacy against XDR *A. baumannii*, consistent with findings from other studies (31, 34). Only a minority of the isolates were susceptible to aminoglycosides (AMI: 12.3%, GEN: 10.8%), reflecting the generally reduced activity of aminoglycosides against XDR *A. baumannii* strains noted in the literature (30, 38). RIF displayed diminished activity against the studied isolates, which contrasts with other reports (33, 35, 36).

The study demonstrated that SUL and FOS, as individual agents, exhibited limited *in vitro* activity against XDR *A. baumannii* strains. However, when these agents were combined, they had a significant synergistic effect on 41.9% of isolates. Mohd Sazlly Lim et al. demonstrate that the SUL/FOS combination exhibits synergistic activity against 74% of carbapenem-resistant *A. baumannii* isolates, with no antagonism observed (39). These findings contrast with the work of Manikal et al.*,* who reported no synergistic activity in SUL/FOS combinations (40). Nevertheless, other studies concur with our observations that FOS, when paired with COL, AMI, or tobramycin, exhibits synergistic effects between 22% and 44% (31, 34, 41). Despite its lack of susceptibility to *A. baumannii*, FOS, when combined with other agents, can enhance the bactericidal activity of the co-administered agent by inhibiting an early stage of bacterial cell wall synthesis, potentially leading to increased uptake of the co-administered agents (39). FOS combined with SUL or other agents may be a promising option for treating XDR *A. baumannii*, but further clinical studies are urgently needed.

While COL and TIG demonstrated potent activity against our isolates, their synergistic effects when combined with SUL were limited, affecting only 6.5% and 12.9% of the isolates, respectively. Moreover, more than 50% of the isolates were indifferent (FICI >1–4) to these combinations, which is notably lower than the synergy rates of 25%–50% reported for SUL/COL combinations in other studies (31, 32, 42). Despite the bactericidal properties of COL, its tendency to promote microbial regrowth can limit the effectiveness of COL combination therapies (43). Previous studies reported that TIG combined with SUL achieves low synergy rates (8%–10%), which is similar to our findings (29, 33, 44). Due to TIG’s bacteriostatic property, XDR *A. baumannii* may potentially develop resistance, leading to limited *in vitro* efficacy when combined with other agents (29, 33).

SIT, in combination with SUL, has shown low to moderate synergistic effects against XDR *A. baumannii*, while additive and indifferent effects were observed at 45.2% and 48.4%, respectively. Previous studies report that SUL/SIT combinations produce an additive effect and indifference in most isolates, consistent with our findings (36, 37). Our study revealed that AMI, MER, and RIF alone exhibited poor inhibitory activity against XDR *A. baumannii*. However, when these agents were combined with SUL, they achieved synergy rates against some isolates. SUL/MER combinations have been reported to display synergistic activities ranging from 30% to 67% against carbapenem-resistant and XDR *A. baumannii* isolates (45–47). Limited data exist for SUL/AMI and SUL/RIF combinations; a few studies indicate synergistic effects in 17% and 4% of cases, respectively (33, 45). Antagonism was infrequently observed in our study: only 1.6%–3.2% of the tested combinations had such effects. Most combinations of SUL with the nine antimicrobial agents resulted in FICIs ranging from >0.5 to 4.0, indicating a reduction in MICs even without synergism.

In this study, a time–kill assay was employed to evaluate the bactericidal efficacy of AMI, FOS, MER, and SUL against three distinct *A. baumannii* isolates. When these agents were tested as a single agent at subinhibitory concentrations (0.25× MICs), they failed to exhibit bactericidal activity against the isolates within 24 hours. This result aligns with previous findings that highlighted the absence of bactericidal effects of individual agents against XDR *A. baumannii* (29, 30, 32, 37, 48). However, other research revealed that increasing the dosage to 1–8× MICs of tested agents results in bacterial eradication, although with subsequent regrowth after 3–8 hours of incubation (29, 32).

This study further investigated the synergistic and bactericidal impacts of various two-agent combinations using the time–kill method. The effectiveness of these combinations depended on the specific drug concentrations and the type and number of *A. baumannii* strains tested. Our analysis revealed reduced growth in two of the isolates after 24 hours of treatment with different two-agent combinations. However, no synergistic effect was observed for the third isolate over the same period.

In our study, the triple combination of AMI, FOS, and MER achieved bactericidal activity against the KO107 isolate after 24 hours. This regimen also demonstrated moderate killing efficacy at 8–10 hours for the SiUA6 isolates. Furthermore, the combination of AMI, FOS, MER, and SUL effectively suppressed the growth of the KO107 and the SiPA14 isolates over a 24 hour period, outperforming the control. The pharmacodynamic actions of FOS and SUL play a pivotal role in these combinations. FOS disrupts the synthesis of peptidoglycan precursors, which are crucial components of the bacterial cell wall. Concurrently, SUL inhibits the transpeptidase activity of penicillin-binding proteins, further impairing cell wall biosynthesis (39). These mechanisms likely facilitate the enhanced penetration of AMI and MER into bacterial cells, thereby augmenting their intracellular efficacy (46, 49). However, the bactericidal effectiveness of three- or four-agent combinations against *A. baumannii* has not been fully explored. The use of three- or four-agent antimicrobial combinations poses a risk of increased adverse drug reactions. Therefore, in-depth pharmacokinetic and pharmacodynamic studies are urgently needed. Such research is essential to determine safe and effective dosing regimens for these complex combinations, especially when targeting XDR *A. baumannii* infections.

*A. baumannii* has demonstrated the capacity to develop resistance during monotherapy, underscoring the potential of SUL for enhancing treatment efficacy against highly resistant strains. Our study highlights the potential of SUL combined with other antimicrobial agents for managing these infections. However, direct translation of *in vitro* results to clinical efficacy should be approached cautiously. The SUL and FOS combination exhibited notable *in vitro* synergistic (41.9%) and additive (40.3%) effects on the isolates, but clinical data supporting the effectiveness of this regimen are not yet available. Additionally, more than 80% of the isolates exhibited a synergistic or additive response to the SUL/MER combination. However, Jean et al. reported lower survival outcomes in patients with XDR *A. baumannii* pneumonia and bacteremia treated with SUL/imipenem than in those receiving TIG/imipenem (50). According to this study, the SUL/COL and SUL/TIG combinations had low synergistic effects, and they exhibited indifferent activity to 54.8% of the isolates. However, a network meta-analysis by Kengkla et al. linked the combination of SUL and COL to increased microbiological success in treating highly resistant *A. baumannii* infections (51). Another meta-analysis suggested that high-dose SUL combined with TIG resulted in greater clinical improvement and less nephrotoxicity than COL-based regimens (52). Our findings indicate that three- or four-agent combination regimens demonstrated sustained bactericidal activity against the two XDR *A. baumannii* isolates. Nonetheless, these *in vitro* results may not fully reflect *in vivo* antimicrobial effectiveness. Consequently, extensive clinical research is needed to validate the use of multi-agent regimens for treating XDR *A. baumannii* infections.

The current study has some limitations. The 62 XDR *A. baumannii* isolates tested *in vitro* may not encompass the full spectrum of resistance mechanisms known for *A. baumannii*. The specific antimicrobial resistance mechanisms and genetic determinants of the study isolates were not analyzed, a factor that could have elucidated the mechanisms underlying the enhanced bactericidal and synergistic activities of combined antimicrobial agents. The reference standard susceptibility testing of various agents, such as FOS, SIT, TIG, and RIF, against *A. baumannii* has not been clearly elucidated. Therefore, the accuracy of the broth microdilution method currently used for these agents should be further evaluated. Moreover, the *in vitro* analysis was restricted to combined agents available at our institution, excluding potentially effective combination agents such as minocycline and cefiderocol for XDR *A. baumannii* infections. Additionally, although the checkerboard method is extensively used, it may not be as comprehensive as the time–kill method for determining synergistic activity, with synergy rates via the checkerboard method generally lower than those obtained through time–kill assays (53). Resource limitations confined the time–kill method to only three XDR *A. baumannii* isolates, potentially limiting the representativeness of the study’s findings. The diverse *in vitro* activities observed across different antimicrobial regimens are likely due to strain-specific variations. Furthermore, results from *in vitro* studies do not reflect the changing drug concentrations *in vivo*. These findings underscore the need for further detailed evaluation using dynamic *in vivo* or animal models and clinical research to fully understand the effectiveness of these antimicrobial combinations.

In conclusion, this study revealed that the activity of SUL combined with available antimicrobial agents had varying degrees of synergistic effects on XDR *A. baumannii* isolates. A small number of isolates exhibited antagonistic responses to SUL-based combinations. Notably, the SUL/FOS/MER/AMI combination regimen displayed sustained bactericidal activity over 24 hours against the two isolates, outperforming the double- and triple-combination regimens. However, the clinical effectiveness of these *in vitro* results, particularly concerning SUL-based combination therapy for XDR *A. baumannii* infections, remains to be validated. Consequently, further clinical investigations are necessary to fully evaluate the risks and benefits of these combination therapies.

## MATERIALS AND METHODS

### Studied isolates and antimicrobial agents

This investigation included 62 clinically isolated strains of XDR *A. baumannii*, collected between 2016 and 2020 and stored at −80°C. XDR *A. baumannii* is defined as the strain that is resistant to nearly all antimicrobial agents and only susceptible to one or two agents (5). These isolates were revived on blood and MacConkey agar plates at 37°C. The strains exhibited growth on MacConkey agar as either lactose non-fermenters (colorless colonies) or glucose oxidizers (slightly pinkish colonies). They tested negative for oxidase, displayed an alkaline/neutral reaction in triple sugar iron slant, and showed positive/negative glucose metabolism in oxidative-fermentative tubes. Notably, these non-motile isolates could grow at 44°C (54). The MICs of AMI, CIP, COL, FOS, GEN, MER, RIF, SIT, SUL, and TIG were determined using the broth microdilution method. FOS susceptibility testing is complicated, as agar dilution is the reference method for the CLSI M100, 2020 (27). However, agar dilution is time-consuming and labor intensive, making it an impractical method for separate testing in our institute. Additionally, the reference standard for FOS susceptibility against *A. baumannii* remains unknown. Therefore, the broth microdilution method was used as a practical susceptibility testing for this agent. Standard powders of these antimicrobial agents were obtained from Glentham Life Sciences Ltd., UK (FOS, SUL, and TIG); Gold Biotechnology Inc., USA (AMI, CIP, COL, GEN, glucose-6-phosphate, and MER); Chem-Impex International Inc., USA (RIF); and Daiichi Sankyo, Japan (SIT). The Siriraj Institutional Review Board approved this study and waived the requirement for subject consent, considering its *in vitro* nature.

### Broth microdilution method and MIC determination

In the broth microdilution process, twofold serial dilutions of each antimicrobial were prepared in cation-adjusted Mueller–Hinton broth (Becton Dickinson, USA). The MIC concentrations used were as follows: AMI, 0.12–256 mg/L; CIP, 0.25–32.0 mg/L; COL, 0.25–32.0 mg/L; FOS, 1–512 mg/L (supplemented with 25 mg/L glucose-6-phosphate); GEN, 0.12–32.0 mg/L; MER, 2–256 mg/L; RIF, 0.12–256 mg/L; SIT, 0.12–32.0 mg/L; SUL, 4–512 mg/L; and TIG, 0.06–8.0 mg/L. The MIC value was the lowest concentration that inhibited visible growth of the XDR *A. baumannii* isolates in accordance with the CLSI guideline M07, 2018 (55). Quality control included *Escherichia coli* American Type Culture Collection (ATCC) 25922 and *Pseudomonas aeruginosa* ATCC 27853. MIC values were interpreted using the CLSI M100, 2020, breakpoints, except for FOS, RIF, SIT, SUL, and TIG, which lack established CLSI breakpoints (27).

### *In vitro* synergy testing via the checkerboard microdilution method

Subsequent to the determination of MICs, a dilution series ranging from 8× to 1/8 MIC for each antimicrobial was prepared using Mueller–Hinton broth. For FOS combinations, the broth was supplemented with 25 mg/L glucose-6-phosphate. SUL was added in a columnar fashion, while co-agents were added in rows. In this study, the synergistic effects of each antimicrobial combination were evaluated for SUL combined with AMI, CIP, COL, FOS, GEN, MER, RIF, SIT, and TIG against all individual 62 XDR *A. baumannii* isolates. Bacterial suspensions at a concentration of 1 × 10⁵ CFU/mL were subsequently introduced, and the cultures were incubated overnight at 37°C (56). The FICI was used to evaluate synergistic interactions between SUL and its co-agents. The FICIs were calculated as follows: (MIC of SUL in combination / MIC of SUL alone) + (MIC of the co-agent in combination / MIC of the co-agent alone), categorizing interactions as synergistic (FICI ≤0.5), additive (>0.5–1.0), indifferent (>1.0–4.0), or antagonistic (FICI >4.0) (57). The checkerboard assays were conducted in duplicate, and average FICIs are reported.

### Time–kill methodology analysis

Selected isolates, representing a range of susceptibilities of tested agents, were assessed using SUL-based combinations via the time–kill method to monitor growth changes quantified as log_10_ CFU/mL over 24 hours (58). Concentrations set at 0.25× MICs for SUL and other antimicrobial agents were used to examine potential synergistic effects at sub-MIC levels. The study using 0.25× MICs aims to determine whether combining two or more antimicrobial agents at sub-MIC levels can produce greater bactericidal activity and overcome resistance mechanisms that develop when bacteria are exposed to higher concentrations of single agents, as reported by several studies (59–61). Viable bacteria were quantified using a 0.1 mL aliquot followed by 10-fold serial dilutions in 0.9 mL of normal saline. Aliquots of 0.1 mL from both the undiluted and diluted samples were plated on trypticase soy agar and incubated at 37°C for 24–48 hours. The minimum colony-count threshold was set at 10 CFU/mL. Time–kill curves representing the bactericidal efficacy of individual or combined antimicrobial agents were plotted at 0, 2, 4, 6, 8, 10, and 24 hours using average log_10_ CFU counts. The bactericidal activity of the drug combinations was defined as a reduction of ≥3 log_10_ CFU/mL over the duration, and the synergistic effect was defined as a decrease of ≥2 log_10_ CFU/mL relative to the most active single agent at the 24 hour mark.

## Data Availability

The datasets generated and/or analyzed during this study are available and can be provided by the corresponding author upon reasonable request.

## SUPPLEMENTAL MATERIAL

Table S1 spectrum.01379-25-s0001.docx MIC data of all study isolates.

ASM does not own the copyrights to Supplemental Material that may be linked to, or accessed through, an article. The authors have granted ASM a non-exclusive, world-wide license to publish the Supplemental Material files. Please contact the corresponding author directly for reuse.
