# Supplementary material for: In vitro activity of sulbactam in combination with other antimicrobial agents against extensively drug-resistant Acinetobacter baumannii
Source: Microbiol Spectr. 2025 Aug 12;13(9):e01379-25. doi: 10.1128/spectrum.01379-25 (PMC12403775; doi:10.1128/spectrum.01379-25)
Supplement: Table S1 — MIC data of all study isolates. [file spectrum.01379-25-s0001.docx]

**Supplementary Table.** The minimal inhibitory concentration (MIC) data of ten antimicrobial agents against all individual XDR *A. baumannii* isolates determined by the broth microdilution method

| **Code ID** | **SUL** | **AMI** | **CIP** | **COL** | **FOS** | **GEN** | **MER** | **RIF** | **SIT** | **TIG** |
| --- | --- | --- | --- | --- | --- | --- | --- | --- | --- | --- |
| SiUA 2 | 64 | 64 | >32 | 1 | 512 | >32 | 128 | 4 | 1 | 0.5 |
| SiPA 5 | 64 | 64 | >32 | 0.5 | >512 | >32 | 128 | 8 | 1 | 1 |
| SiUA 3 | 64 | 64 | >32 | 0.5 | >512 | >32 | 128 | 4 | 2 | 0.5 |
| PK/PD 76 | 128 | 64 | >32 | 1 | 256 | >32 | 128 | 8 | 2 | 1 |
| SiPA 15 | 64 | 64 | >32 | 1 | >512 | >32 | 32 | 128 | 1 | 1 |
| SiPA 13 | 64 | 64 | >32 | 2 | 256 | >32 | 64 | 64 | 1 | 1 |
| SiUA 10 | 32 | 64 | >32 | 2 | >512 | >32 | 32 | 8 | 1 | 1 |
| SiUA 18 | 32 | 0.5 | >32 | 2 | 512 | 0.25 | 128 | 8 | 1 | 0.5 |
| SiUA 19 | 64 | 4 | >32 | 2 | 512 | >32 | 128 | 8 | 1 | 1 |
| SiUA 8 | 32 | 64 | >32 | 1 | 512 | >32 | 64 | 8 | 4 | 1 |
| SiUA 9 | 32 | 64 | >32 | 1 | 512 | >32 | 32 | 4 | 1 | 1 |
| SiPA 7 | 64 | 64 | >32 | 1 | 512 | >32 | 128 | 64 | 1 | 1 |
| SiUA 1 | 64 | 64 | >32 | 0.5 | >512 | >32 | 128 | 8 | 4 | 1 |
| KMS 97 | 64 | 64 | >32 | 1 | 256 | >32 | 64 | 8 | 2 | 1 |
| SiUA 11 | 64 | 64 | >32 | 1 | 512 | >32 | 128 | 8 | 4 | 1 |
| KL35 | 64 | 64 | >32 | 1 | >512 | >32 | 64 | 128 | 4 | 1 |
| KK 107 | 64 | 64 | >32 | 1 | 512 | >32 | 128 | 4 | 1 | 2 |
| KL 069 | 32 | 2 | >32 | 0.5 | 512 | 2 | 64 | 8 | 1 | 0.5 |
| SWS 089 | 64 | 2 | >32 | 2 | 512 | >32 | 64 | 4 | 1 | 1 |
| KMS 84 | 64 | 64 | >32 | 1 | 512 | >32 | 64 | 1 | 2 | 1 |
| KMS 86 | 256 | 64 | >32 | 1 | 256 | >32 | 256 | 128 | 2 | 0.5 |
| KMS 91 | 64 | 64 | >32 | 1 | 256 | >32 | 128 | 4 | 1 | 0.5 |
| SiUA 13 | 64 | 64 | >32 | 1 | 512 | >32 | 64 | 128 | 4 | 1 |
| KK 95 | ≤4 | 4 | 0.5 | 1 | 256 | 0.25 | 32 | 8 | ≤0.12 | 0.12 |
| SiUA 23 | 16 | 64 | >32 | 1 | >512 | >32 | 64 | 2 | 1 | 0.25 |
| SiPA 28 | 64 | 64 | >32 | 0.5 | 256 | >32 | 128 | 128 | 1 | 1 |
| SiPA 4 | 64 | 64 | >32 | 1 | >512 | >32 | 128 | 128 | 4 | 1 |
| SWS 93 | 32 | 64 | >32 | 1 | 512 | >32 | 64 | 8 | 4 | 1 |
| SWS 98 | 32 | 64 | >32 | 1 | 512 | >32 | 128 | 8 | 4 | 1 |
| SiUA 20 | 64 | 2 | >32 | 1 | 512 | >32 | 128 | 8 | 1 | 1 |
| KMU 98 | 16 | 64 | >32 | 1 | 256 | >32 | 64 | 4 | 1 | 0.5 |
| SWS 81 | 32 | 64 | >32 | 1 | 256 | >32 | 128 | 4 | 2 | 1 |
| SiUA 16 | 128 | 1 | >32 | 1 | 512 | 1 | >256 | 8 | 2 | 1 |
| SiPA 14 | 128 | 64 | >32 | 1 | 512 | >32 | 128 | 8 | 1 | 1 |
| KMU 94 | 64 | 64 | >32 | 1 | 256 | >32 | 256 | 128 | 2 | 1 |
| SiUA 6 | 64 | 1 | >32 | 1 | 512 | 2 | 256 | 8 | 1 | 1 |
| KK 102 | 8 | 64 | >32 | 1 | 512 | >32 | 32 | 128 | 1 | 1 |
| SiPA 11 | 64 | 64 | >32 | 1 | 256 | >32 | 128 | 128 | 1 | 1 |
| SiPA 12 | 64 | 64 | >32 | 1 | >512 | >32 | 64 | 8 | 4 | 1 |
| KL 72 | 32 | 64 | >32 | 2 | 256 | >32 | 128 | 8 | 2 | 1 |
| KK 104 | 32 | 64 | >32 | 1 | 256 | >32 | 32 | 8 | 2 | 1 |
| SiUA 5 | 32 | 64 | >32 | 1 | 512 | >32 | 64 | 8 | 4 | 1 |
| SiPA 10 | 64 | 64 | >32 | 1 | >512 | >32 | 128 | 64 | 2 | 1 |
| KK 91 | 64 | 128 | >32 | 1 | 256 | >32 | 128 | 4 | 4 | 2 |
| SiUA 14 | 64 | 64 | >32 | 1 | 512 | >32 | 128 | 8 | 4 | 1 |
| SiPA 2 | 128 | 64 | >32 | 1 | >512 | >32 | 128 | 8 | 4 | 1 |
| SiUA 15 | 32 | 64 | >32 | 0.5 | 512 | >32 | 128 | 4 | 1 | 0.5 |
| KO 106 | 64 | 64 | >32 | 1 | 512 | >32 | 256 | 2 | 1 | 1 |
| KO 107 | 64 | 64 | >32 | 1 | 512 | >32 | 256 | 32 | 4 | 4 |
| KO 109 | 64 | 64 | >32 | 1 | 256 | >32 | 256 | 64 | 1 | 1 |
| KO 110 | 64 | 64 | >32 | 1 | 64 | >32 | 128 | 128 | 2 | 1 |
| KO 112 | 64 | 64 | >32 | 0.5 | 512 | >32 | 128 | 8 | 2 | 0.5 |
| KO 113 | 32 | 64 | >32 | 1 | 512 | >32 | 128 | 8 | 2 | 1 |
| KO 116 | 128 | 64 | >32 | 0.5 | 512 | >32 | 256 | 32 | 1 | 1 |
| KO 121 | 32 | 2 | 32 | 1 | 256 | 0.5 | 64 | 8 | 1 | 1 |
| KO 122 | 64 | 64 | >32 | 1 | 256 | >32 | 16 | 128 | 1 | 0.5 |
| KO 126 | 64 | 32 | >32 | 0.5 | 256 | >32 | 128 | 4 | 1 | 0.5 |
| KO 127 | 128 | 0.25 | 32 | 1 | 256 | ≤ 0.12 | 64 | 8 | 0.5 | 0.25 |
| KO 128 | 32 | 64 | >32 | 1 | 512 | >32 | 64 | 4 | 1 | 0.5 |
| KO 131 | 32 | 64 | >32 | 1 | 256 | >32 | 64 | 8 | 1 | 1 |
| KO 132 | 32 | 64 | >32 | 0.5 | 256 | >32 | 64 | 8 | 1 | 1 |
| KO 129 | 32 | 64 | >32 | 1 | 256 | >32 | 128 | 8 | 2 | 0.5 |
| **N 62** |  |  |  |  |  |  |  |  |  |  |
| **MIC Range** | ≤4-256 | 0.25-128 | 0.5->32 | 0.5-2 | 64->512 | ≤0.12->32 | 16->256 | 1-128 | ≤0.12-4 | 0.12-4 |
| **MIC50** | 64 | 64 | >32 | 1 | 512 | >32 | 128 | 8 | 1 | 1 |
| **MIC90** | 128 | 64 | >32 | 1 | >512 | >32 | 256 | 128 | 4 | 1 |

Abbreviations: AMI, amikacin; CIP, ciprofloxacin; COL, colistin; FOS, fosfomycin; GEN, gentamicin; MER, meropenem; RIF, rifampicin; SIT, sitafloxacin; SUL, sulbactam; TIG, tigecycline
